# Adaptive Neural Network Motion Control of Manipulators with Experimental Evaluations

**DOI:** 10.1155/2014/694706

**Published:** 2014-01-19

**Authors:** S. Puga-Guzmán, J. Moreno-Valenzuela, V. Santibáñez

**Affiliations:** ^1^Instituto Tecnológico de Tijuana, Boulevard. Industrial S/N, 22510 Tijuana, BC, Mexico; ^2^Instituto Politécnico Nacional-CITEDI, Avenida del Parque 1310, Mesa de Otay, 22510 Tijuana, BC, Mexico; ^3^Instituto Tecnológico de La Laguna, Boulevard Revolución y Cuahtémoc, 27000 Torreón, COAH, Mexico

## Abstract

A nonlinear proportional-derivative controller plus adaptive neuronal network compensation is proposed. With the aim of estimating the desired torque, a two-layer neural network is used. Then, adaptation laws for the neural network weights are derived. Asymptotic convergence of the position and velocity tracking errors is proven, while the neural network weights are shown to be uniformly bounded. The proposed scheme has been experimentally validated in real time. These experimental evaluations were carried in two different mechanical systems: a horizontal two degrees-of-freedom robot and a vertical one degree-of-freedom arm which is affected by the gravitational force. In each one of the two experimental set-ups, the proposed scheme was implemented without and with adaptive neural network compensation. Experimental results confirmed the tracking accuracy of the proposed adaptive neural network-based controller.

## 1. Introduction

Robust control consists in designing control strategies by using little information of the system model and considering that the system may be affected by bounded disturbances. Robust controllers can be designed to satisfy either the regulation goal or the trajectory tracking objective. Thus, in the last years there has been mathematical and practical interest in studying robust control architectures. In this class of controllers, adaptive neural networks have been used in the design of robust controllers for electromechanical systems.

Neural networks can be used in the control of unknown systems without requirements for linearity in the system parameters. Neural networks exhibit the universal approximation property which allows approximating unknown linear and nonlinear functions [1].

In the following, we provide a literature review on application of neural networks for robot motion control. Selmic and Lewis [2] proposed a dynamical inversion compensation scheme by using a backstepping technique with neural networks, which was applied to mechanical systems. Kwan et al. [3] proposed a class of neural network robust controllers showing global asymptotic stability of tracking errors and boundedness of neural network weights. Lewis et al. [1] provided in manner of survey a study of the application of neural networks in the compensation of actuator nonlinearities. In [4] a robust controller with a nonlinear two-layer neural network structure was proposed for control of four-axis SCARA robot manipulator. The error trajectories are proven to be uniformly ultimately bounded. Yu and Li [5] propose a PD-type controller plus neural network compensation of the uncertainties and velocity estimation is achieved by using a high-gain observer. Stability is proven using Lyapunov-based analysis. Wang et al. [6] proposed a neural network-based motion controller in task space. The controller is addressed as two-loop cascade control scheme. The inner loop implements a velocity servo loop at the robot joint level using a radial basis function network with a proportional-integral controller. In [7] an adaptive neural network algorithm is developed for rigid-link electrically driven robot systems. The controller is developed in a constructive form and a rigorous stability analysis is also provided. In Moreno-Armendáriz et al. [8], an indirect adaptive control using hierarchical fuzzy CMAC neuronal network for the ball and plate system was introduced. The proposed controller was validated by means of numerical simulations and experiments.

Fateh and Alavi [9] introduced a new scheme for the impedance control of an active suspension system. The control was achieved through two interior loops which are force control of the actuator by feedback linearization and fuzzy control loop. The work proposed by Liu et al. in [10, 11] addressed the problem of control of manipulators with saturated input. In particular, new controllers based on fuzzy self-tuning of the proportional and derivative gains were proposed. Although the controllers in [9–11] are based in fuzzy logic, it has been proven in [12] that fuzzy logic systems and feedforward neural networks are equivalent in essence.

In Sun et al. [13], a robust tracking control for robot manipulators in the presence of uncertainties and disturbances was proposed. A neural network-based sliding mode adaptive control was designed to ensure trajectory tracking by the robot manipulator. In Hernandez et al. [14], a neural PD with a second order sliding mode compensation technique was introduced. The scheme is able to guarantee asymptotic convergence of the error trajectories. In [15], in order to deal with actuator and model nonlinearities, a neural network-based controller was addressed. The scheme was experimentally tested in real time showing the advantages of the neural network. In [16], neural network contouring control using a Zebra-Zero robotic manipulator was presented. The paper in [17] used two modified optimal controllers based on neural networks. The closed-loop system trajectories were studied in a rigorous form although validation was presented with simulations. Other novel approaches of neural networks are presented in [18, 19].

Experimental evidence that neural networks are efficient in the control of a mechanical systems has been provided in [8, 15], for example. However, existing literature reveals a gap in the experimental evaluation of new controllers since most of the published works only consider numerical simulations to assess the performance of the proposed controllers.

In the present work a different approach is taken. In the theoretical part of our work, a neural network is used to approach the desired torque as a function of the desired joint position trajectory. The inner and outer weights of the neural network are adapted on-line by using an update law coming from a Lyapunov-like analysis of the closed-loop system trajectories. In addition, an extensive real-time implementation study in two different experimental set-ups has also been carried out. We prove by means of the experimental tests that the neural network compensation is really effective to reduce the joint tracking error. The real-time experiments show that an excellent tracking accuracy can be obtained by using adaptive neural network compensation plus a “small amount” of nonlinear PD control compensation. In summary, the contribution of this paper is twofold:

1. a new nonlinear PD controller plus adaptive neural network compensation,
2. a real-time experimental study in two different experimental set-ups.

Our approach is based on the adaptation of the weights of a neural network that only depends on the desired signals of joint position, velocity, and acceleration. In other words, the neural network used in the controller approaches the desired torque. In addition to the adaptive neural network feedforward compensation, the proposed controller is equipped with nonlinear PD terms, which are motivated by the convergence analysis. We prove that by using the proposed controller; the position and velocity error trajectories converge to zero, while the adapted neural network signals remain uniformly bounded. It is noteworthy that the proposed neural network controller resembles the PD control with feedforward compensation for robot manipulators, whose global asymptotical convergence proof was reported in [20, 21].

The proposed controller is tested in real time in a horizontal two-degree-of-freedom direct-driven arm and in a vertical single-link arm, which is affected by the gravitational force. In both experimental set-ups, the new scheme is implemented with and without neural network adaptation. The experimental results show that the tracking performance is drastically improved by using the new controller with the adaptive neural network feedforward compensation.

The difference of this document with respect to our previous work in [22] is that here the study of closed-loop trajectories is rigorously presented and experimental validation of the proposed scheme is evaluated in a detailed form.

The present document is organized as follows. Mathematical preliminaries, the robot model, and the control goal are given in Section 2. The proposed controller is discussed in Section 3.  Section 4 is devoted to the real-time experimental result, while some concluding observations are given in Section 5.

## 2. Mathematical Preliminaries, Robot Model, and Control Goal

The notations *λ*_min_{*A*} and *λ*_max_{*A*} denote the minimum and maximum eigenvalues of a symmetric positive definite matrix *A* ∈ ℝ^*n*×*n*^, respectively. $||x||=\sqrt{x^{T}x}$ stands for the norm of vector **x** ∈ ℝ^*n*^. $||B||\;=\sqrt{\lambda_{\max}\{B^{T}B\}}$ stands for the induced norm of a matrix *B*(**x**) ∈ ℝ^*m*×*n*^ for all **x** ∈ ℝ^*n*^.

Given *A* ∈ ℝ^*n*×*m*^ the Frobenius norm is defined [1, 23] by

(1) $$\begin{array}{c} {||A||}_{F}^{2}=\mathrm{Tr}\left(A^{T}A\right)=\underset{i,j}{\sum }a_{ij}^{2}. \end{array}$$

Other properties are

(2) 〈A,B〉F=Tr(ATB),|〈A,B〉F|≤||A||F||B||F.

### 2.1. Properties on Hyperbolic Functions

Some properties on hyperbolic functions will be used. See [24, 25], where the cited properties are used in the design and analysis of controllers for mechanical systems. The tangent hyperbolic function is defined as

(3) $$\begin{array}{c} \tanh\left(x\right)=\frac{e^{x}-{\text{e}}^{-x}}{e^{x}+e^{-x}}, \end{array}$$

where *x* ∈ ℝ. The tangent hyperbolic function can be arranged in a vector in the following way:

(4) $$\begin{array}{c} \tanh\left(z\right)={\left[\begin{array}{c} \tanh\left(z_{1}\right),\ldots ,\tanh\left(z_{n}\right) \end{array}\right]}^{T}, \end{array}$$

and the following properties are accomplished by tanh(**z**).

(a) For all **z** ∈ ℝ^*n*^, the Euclidean norm of tanh(**z**) satisfies
  (5) ||tanh(z)||≤||z||,||tanh(z)||≤n.
(b) The time derivative of tanh(**z**) is given by
  (6) $$\begin{array}{c} \frac{d}{dt}\tanh\left(z\right)=\text{Sec}{\text{h}}^{2}\left(z\right)\dot{z}, \end{array}$$
 where Sech^2^(**z**) = diag{sech^2^(*z*_1_),…, sech^2^(*z*_*n*_)} and
  (7) $$\begin{array}{c} \text{Sech}\left(x\right)=\frac{2}{e^{x}+e^{-x}}=\frac{1}{\cosh\left(x\right)}. \end{array}$$
(c) The maximum eigenvalue of the matrix sech^2^(**x**) is one for all **z** ∈ ℝ^*n*^; that is,
  (8) $$\begin{array}{c} \lambda_{\max}\left\{\text{sec}{\text{h}}^{2}\left(z\right)\right\}=1. \end{array}$$
(d) Finally, the property
  (9) $$\begin{array}{c} \sqrt{\overset{n}{\underset{i=1}{\sum }}\ln\left(\cosh\left(z_{i}\right)\right)}\geq \frac{1}{\sqrt{2}}||\tanh\left(z\right)||,\;\forall z\in {\mathbb{R}}^{n}, \end{array}$$
 holds.

### 2.2. Neural Networks

Let us recall the universal approximation property of the neural networks [1, 3]. A function **f**(**x**) : ℝ^*N*+1^ → ℝ^*n*^ can be approximated by

(10) $$\begin{array}{c} f\left(x\right)=W^{T}\sigma \left(V^{T}x\right)+\epsilon ,\;\forall x\in {\mathbb{R}}^{N+1}, \end{array}$$

where **x** ∈ ℝ^*N*+1^ is the vector of input signals to the neural network, *V* ∈ ℝ^(*N*+1)×*L*^ and *W* ∈ ℝ^*L*×*n*^ are the input and output ideal weights, respectively, *L* is the number of neurons in the hidden layer, *N* + 1 is the number of input signals to the neural network, **σ** ∈ ℝ^*L*^ is the activation function in the hidden layer, and **ϵ** ∈ ℝ^*n*^ is the approximation error with

(11) $$\begin{array}{c} \left|\epsilon_{i}\right|\leq \phi ,\;i=1,\ldots ,n, \end{array}$$

where *ϕ* > 0.

The output of an activation function, *σ*_*i*_ : ℝ → ℝ, *i* = 1,…, *L*, is used to define the output signal of a neuron from a modified combination of its input signals by compressing the signal. The function *σ*_*i*_ is usually between the values 0 ≤ *σ*_*i*_ ≤ 1 or valued such that −1 ≤ *σ*_*i*_ ≤ 1 is satisfied.

In this paper, we have used as activation function *σ*_*i*_ the hyperbolic tangent function. Therefore, by defining **z** = *V* ^*T*^ **x**,

(12) $$\begin{array}{c} \sigma \left(z\right)={\left[\begin{array}{c} \tanh(z_{1}),\ldots ,\tanh(z_{L}) \end{array}\right]}^{T}. \end{array}$$

### 2.3. Robot Dynamics

The dynamics in joint space of a serial-chain *n*-link robot manipulator considering the presence of friction at the robot joints can be written as [26, 27]

(13) $$\begin{array}{c} M\left(q\right)\ddot{q}+C\left(q,\dot{q}\right)\dot{q}+g\left(q\right)+F_{v}\dot{q}=\tau , \end{array}$$

where **q** ∈ ℝ^*n*^ is a vector of joint positions, *M*(**q**) ∈ ℝ^*n*×*n*^ is the symmetric positive definite inertia matrix, $C(q,\dot{q})\dot{q}\in {\mathbb{R}}^{n}$ is the vector of centripetal and Coriolis torques, **g**(**q**) ∈ ℝ^*n*^ is the vector of gravitational torques, *F*_*v*_ ∈ ℝ^*n*×*n*^ is a diagonal positive definite matrix containing the viscous friction coefficients of each joint, and **τ** ∈ ℝ^*n*^ is the vector of torques input.

The dynamics of *n*-link robotic manipulator expressed in (13) has the following properties, which hold for rigid-link revolute joint manipulators [26–28].

**Property 1**

The inertia matrix *M*(**q**) is symmetric and positive definite; that is,

(14) $$\begin{array}{c} \lambda_{\min}\left\{M\left(q\right)\right\}{||x||}^{2}\leq x^{T}M\left(q\right)x\leq \lambda_{\max}\left\{M\left(q\right)\right\}{||x||}^{2}. \end{array}$$

**Property**

Assuming that the robot has revolute joints, the vector *C*(**q**, **x**)**y** satisfies the bound

(15) $$\begin{array}{c} ||C\left(q,x\right)y||\leq k_{C1}||x||||y||,\;\forall q,x,y\in {\mathbb{R}}^{n}, \end{array}$$

where *k*_*C*1_ > 0.

**Property 3**

Assume that the centrifugal and Coriolis torque matrix $C(q,\dot{q})$ is computed by means of the so-called Christoffel symbols of the first kind. Then,

(16) $$\begin{array}{c} x^{T}\left[\dot{M}\left(q\right)-2C\left(q,\dot{q}\right)\right]x=0,\;\forall x,q,\dot{q}. \end{array}$$

Besides,

(17) $$\begin{array}{c} \dot{M}\left(q\right)=C\left(q,\dot{q}\right)+C{\left(q,\dot{q}\right)}^{T}. \end{array}$$

**Property 4**

The so-called residual dynamics [29, 30] is defined by

(18) h(t,e,e˙)=[M(qd)−M(q)]q¨d+[C(qd,q˙d)−C(q,q˙)]q˙d+g(qd)−g(q),

where **e** will be defined later and **q**_*d*_(*t*) is the desired joint position trajectory assumed to be bounded together with its first and second time derivative. The residual dynamics (18) satisfies the following inequality [27]:

(19) $$\begin{array}{c} ||h\left(t,e,\dot{e}\right)||\leq k_{h1}||\dot{e}||+k_{h2}||\tanh\left(\gamma e\right)||, \end{array}$$

where *k*_*h*1_ and k_*h*1_ are sufficiently large strictly positive constants that depend on the robot model parameters and *γ* > 0.

### 2.4. Control Goal

Let us define the joint position tracking error as

(20) $$\begin{array}{c} e\left(t\right)=q_{d}\left(t\right)-q\left(t\right), \end{array}$$

where **q**_*d*_(*t*) ∈ ℝ^*n*^ denotes the desired joint position trajectory. The estimated weight errors are defined as

(21) $$\begin{array}{c} \begin{array}{c} \tilde{W}=W-\hat{W},\\ \tilde{V}=V-\hat{V}, \end{array} \end{array}$$

where *V* ∈ ℝ^[*N*+1]×*L*^ are the ideal input weights, $\hat{V}\in {\mathbb{R}}^{[N+1]\times L}$ is an estimation of the input weights, *W* ∈ ℝ^*L*×*n*^ are the ideal output weights, and $\hat{W}\in {\mathbb{R}}^{L\times n}$ is an estimation of the output weights.

The desired time-varying trajectory **q**_*d*_(*t*) is assumed to be three times differentiable and bounded for all time *t* ≥ 0 in the sense

(22) $$\begin{array}{c} \begin{array}{c} ||q_{d}\left(t\right)||\leq \mu_{1},\\ ||{\dot{q}}_{d}\left(t\right)||\leq \mu_{2},\\ ||{\ddot{q}}_{d}\left(t\right)||\leq \mu_{3}, \end{array} \end{array}$$

where *μ*_1_, *μ*_2_, and *μ*_3_ denote known positive constants.

The control problem consists in designing a controller **τ**(*t*) and update laws for the estimated weights $\hat{V}(t)$ and $\hat{W}(t)$ such that the signals **e**(*t*), $\dot{e}(t)$, $\tilde{W}(t)$, and $\tilde{V}(t)$ are uniformly bounded. In addition, the limit

(23) $$\begin{array}{c} \underset{t\to \infty }{\lim}\left[\begin{array}{c} e\left(t\right)\\ \dot{e}\left(t\right) \end{array}\right]=0 \end{array}$$

should be satisfied.

## 3. Proposed Adaptive Neuronal Network Compensation Controller

The proposed controller has a structure of a nonlinear PD controller plus adaptive neural network feedforward compensation. The design of the controller departs from the assumption that the desired torque can be approached by a neural network and then it can be estimated on-line by means of proper adaptation laws for the input and output neural network weights.

### 3.1. Proposed Scheme

The development of the proposed approach is presented in a constructive form. First, let us consider the robot dynamics (13) evaluated along the desired position **q**_*d*_ ∈ ℝ^*n*^ such that the desired torque **τ**_*d*_ ∈ ℝ^*n*^ can be founded by

(24) $$\begin{array}{c} M\left(q_{d}\right){\ddot{q}}_{d}+C\left(q_{d},{\dot{q}}_{d}\right){\dot{q}}_{d}+g\left(q_{d}\right)+F_{v}{\dot{q}}_{d}=\tau_{d}, \end{array}$$

by combining (13) and (24) and using the tracking error defined in (20) the following equation is obtained:

(25) $$\begin{array}{c} M\left(q\right)\ddot{e}+C\left(q,\dot{q}\right)\dot{e}+h\left(t,e,\dot{e}\right)+F_{v}\dot{e}=\tau_{d}-\tau , \end{array}$$

where $h(t,e,\dot{e})\in {\mathbb{R}}^{n}$ is the so-called residual dynamics [29, 30], defined by (18).

By using the universal approximation property of the neural networks in (10), the desired torque in (24) can be approached as

(26) $$\begin{array}{c} \tau_{d}=W^{T}\sigma \left(V^{T}x_{d}\right)+\epsilon , \end{array}$$

where

(27) $$\begin{array}{c} x_{d}={\left[q_{d}^{T}\;{\dot{q}}_{d}^{T}\;{\ddot{q}}_{d}^{T}\;1\right]}^{T}\in {\mathbb{R}}^{N+1} \end{array}$$

is the vector of input signals to the neural network. Notice that in this case *N* = 3*n*.

Now, we are in position to introduce the following tracking controller:

(28) $$\begin{array}{c} \tau ={\hat{W}}^{T}\sigma \left({\hat{V}}^{T}x_{d}\right)+K_{p}\tanh\left(\gamma e\right)+K_{d}\dot{e}+\Delta \mathrm{sign}\left(r\right), \end{array}$$

where *K*_*p*_, *K*_*d*_, and Δ are diagonal positive definite matrices, *γ* is a positive scalar, $\hat{V}$ is the estimated input weight, $\hat{W}$ is the estimated output weight,

(29) r=e˙+αtanh(γe),

(30) sign(r)=[sign(r1)  ⋯  sign(rn)]T∈ℝn,

with

(31) $$\begin{array}{c} \mathrm{sign}\left(x\right)=\{\begin{array}{cc} 1, & \text{for}\;x>0,\\ 0, & \text{for}\;x=0,\\ -1, & \text{for}\;x<0. \end{array} \end{array}$$

The proposed update laws for the estimated input and output weights, denoted as $\hat{V}$ and $\hat{W}$, respectively, are

(32) V^˙=RxdrTW^σ^′,

(33) W^˙=Fσ^rT−Fσ^′V^TxdrT,

where *R* ∈ ℝ^[*N*+1]×[*N*+1]^ and *F* ∈ ℝ^*L*×*L*^ are positive definite matrices, $\hat{\sigma }=\sigma ({\hat{V}}^{T}x_{d})$ and

(34) $$\begin{array}{c} {\hat{\sigma }}^{\prime }=\frac{\partial \sigma \left(x\right)}{\partial x}, \end{array}$$

with ${\hat{V}}^{T}x_{d}$.

### 3.2. Closed-Loop System Derivation

By substituting (26) and (28) in (25), the equation

(35) M(q)e¨+C(q,q˙)e˙+h(t,e,e˙)+Fve˙  =−Kptanh(γe)−Kde˙   −Δsign(r)+WTσ(VTxd)−W^Tσ(V^Txd)+ϵ

is obtained. The weight error is defined as in (21) and

(36) $$\begin{array}{c} \tilde{\sigma }=\sigma -\hat{\sigma }=\sigma \left(V^{T}x_{d}\right)-\sigma \left({\hat{V}}^{T}x_{d}\right). \end{array}$$

Multilayered neural networks are nonlinear in the weights *V* and Taylor's series can be used to approximate the activation function **σ**. Thus,

(37) $$\begin{array}{c} \sigma \left(V^{T}x_{d}\right)=\sigma \left({\hat{V}}^{T}x_{d}\right)+\sigma^{\prime }\left({\hat{V}}^{T}x_{d}\right){\tilde{V}}^{T}x_{d}+O{\left({\tilde{V}}^{T}x_{d}\right)}^{2}, \end{array}$$

where $O{\left({\tilde{V}}^{T}x_{d}\right)}^{2}$ represents the higher order terms. Approximation of activation function via Taylor's series has been used in, for example, [1, 3]. Therefore, substituting (21) and (37) in (35) and simplifying, we obtain the following expression:

(38) Me¨=−h−Ce˙−Kptanh(γe)−[Kd+Fv]e˙ −Δsign(r)+W^Tσ^′V~Txd−W~Tσ^′V^Txd +W~Tσ+ω(t),

where *M* = *M*(**q**), $h=h(e,\dot{e})$, $C=C(q,\dot{q})$, and

(39) $$\begin{array}{c} \omega \left(t\right)={\tilde{W}}^{T}{\hat{\sigma }}^{\prime }V^{T}x_{d}+WO^{2}+\epsilon , \end{array}$$

with $O^{2}=O{\left({\tilde{V}}^{T}x_{d}\right)}^{2}$.

Finally, the dynamics of ${\left[e^{T}\;{\dot{e}}^{T}\right]}^{T}\in {\mathbb{R}}^{2n}$ is given by

(40) ddte=e˙,ddte˙=M−1[−h−Ce˙−Kptanh(γe)−[Kd+Fv]e˙  −Δsign(r)+W^Tσ^′V~Txd−W~Tσ^′V^Txd  +W~Tσ^+ω(t)].

By using the definition of the weight errors (21), we can rewrite the proposed update laws (32)-(33) as

(41) ddtV~=−RxdrTWσ^′+RxdrTW~σ^′,ddtW~=−Fσ^rT+Fσ^′VTxdrT−Fσ^′V~TxdrT.

The overall closed-loop system is given by (40) and (41).

### 3.3. Convergence Analysis

The assumption

(42) $$\begin{array}{c} k_{\omega }\geq ||\omega \left(t\right)||,\;\forall t\geq 0, \end{array}$$

with *k*_*ω*_ > 0 and **ω**(*t*) defined in (39), will be used in the next.

Concerning the trajectories of the closed-loop system (40)-(32), we have the following result.

**Proposition 1**

One assumes that the desired trajectory **q**_*d*_(*t*) is bounded as (22). Then, provided that *λ*_min_{*K*_*p*_}, *λ*_min_{*K*_*d*_} and *λ*_min_{Δ} are sufficiently large, there always exist strictly positive constants *α*_max_* and *α*_min_* such that

(43) $$\begin{array}{c} \alpha_{\min}^{*}<\alpha <\alpha_{\max}^{*} \end{array}$$

guarantees that the trajectories **e**(*t*), $\dot{e}(t)$, $\tilde{W}(t)$, and $\tilde{V}(t)$ of the overall closed-loop system (40)-(32) are uniformly bounded. In addition, the limit

(44) $$\begin{array}{c} \underset{t\to \infty }{\lim}\left[\begin{array}{c} e\left(t\right)\\ \dot{e}\left(t\right) \end{array}\right]=0 \end{array}$$

is satisfied.

**Proof**

Let us consider the function

(45) U(t,e,e˙)=12e˙TMe˙+∑i=1nKpiγ−1ln(cosh(γei))+αtanh(γe)TMe˙+12Tr(W~TF−1W~)+12Tr(V~TR−1V~),

which is positive definite in terms of the state space of the closed-loop system (40)-(32).

By using property (9), a lower bound on $U\left(t,e,\dot{e}\right)$ can be computed as follows:

(46) U(t,e,e˙)≥ηT[12λmin{M}−α2λmax{M}−α2λmax{M}γ−12λmin{Kp}]︸Pη   +12Tr(W~TF−1W~)+12Tr(V~TR−1V~),

with $\eta ={\left[||\dot{e}||\;||\tanh(\gamma e)||\right]}^{T}$.

If *P* is positive definite, then the function $U\left(t,e,\dot{e}\right)$ is globally positive definite and radially unbounded. By using Sylvester's Theorem, the sufficient and necessary condition for *P* to be positive definite is

(47) $$\begin{array}{c} 0<\alpha <\frac{\sqrt{\gamma^{-1}\lambda_{\min}\left\{K_{p}\right\}\lambda_{\min}\left\{M\right\}}}{\lambda_{\max}\left\{M\right\}}. \end{array}$$

Next step in the proof is to compute the time derivative of $U\left(t,e,\dot{e}\right)$ along the closed-loop system trajectories (40) and (32). Thus, we have that

(48) U˙(t,e,e˙)=−e˙T[Kd+Fv]e˙−rTh+rT[ω−Δsign(r)]+αγe˙TMSech2(γe)e˙+αtanh(γe)TCTe˙−αtanh(γe)T[Kd+Fv]e˙−αtanh(γe)TKptanh(γe),

which was obtained thanks to the property (6), the robot model properties (16)-(17), and the facts

(49) Tr(W~Tσ^rT)=Tr(rTW~Tσ^),Tr(W~Tσ^′V^TxdrT)=Tr(rTW~Tσ^′V^Txd),Tr(V~TxdrTW^Tσ^′)=Tr(rTW~Tσ^′V~Txd).

An upper bound on each term of $\dot{U}\left(t,e,\dot{e}\right)$ is computed as follows:

(50) −e˙T[Kd+Fv]e˙≤−λmin{Kd+Fv}||e˙||,−rTh≤αkh2||tanh(γe)||2+[αkh1+kh2]||tanh(γe)||||e˙||+kh1||e˙||2,rT[ω−Δsign(r)]≤−[λmin{Δ}−kω]∑i=1n|ri|,αγe˙TM Sech2(γe)e˙≤αγλmax{M}||e˙||2,αtanh(γe)TCTe˙≤αkC1μ2||tanh(γe)||||e˙||+αkC1n||e˙||2,−αtanh(γe)T[Kd+Fv]e˙  ≤αλmax{Kd+Fv}||tanh(γe)||||e˙||,   −αtanh(γe)TKptanh(γe)  ≤−αλmin{Kp}||tanh(γe)||2,

where the property of the residual dynamics **h** in (19), assumption (42), property (8), property (15), and the fact that $||\dot{q}||\leq \mu_{2}+||\dot{e}||$, with *μ*_2_ defined in (22), were used.

With the computed bounds on each term of $\dot{U}(t,e,\dot{e})$, we can write

(51) $$\begin{array}{c} \dot{U}\left(t,e,\dot{e}\right)\leq -p_{1}^{T}Q_{1}p_{1}-\left[\lambda_{\min}\left\{\Delta \right\}-k_{\omega }\right]\overset{n}{\underset{i=1}{\sum }}\left|r_{i}\right|, \end{array}$$

where

(52) p1=[||tanh(γe)||||e˙||],

(53) Q1=[αa−12[αd+e]−12[αd+e]b−αc],

with

(54) a=λmin{Kp}−kh2,b=λmin{Kd+Fv}−kh1,c=γλmax{M}+kC1n,d=kh2+kC1μ2+λmax{Kd+Fv},e=kh1.

It is easy to observe that if *Q*_1_ in (53) is positive definite and if

(55) $$\begin{array}{c} \lambda_{\min}\left\{\Delta \right\}>k_{\omega }, \end{array}$$

with *k*_*ω*_ defined in (42), then the function $\dot{U}\left(t,e,\dot{e}\right)$ is negative semidefinite. Besides, notice that the matrix *Q*_1_ in (53) can be rewritten as

(56) $$\begin{array}{c} Q_{1}=Q_{a}+Q_{b}, \end{array}$$

where

(57) Qa=[12αa−12αd−12αd12[b−2αc]],Qb=[12αa−12e−12e12b].

If *Q*_*a*_ and *Q*_*b*_ are positive definite then *Q*_1_ is also positive definite. By applying Sylvester's criterion, the matrix *Q*_*a*_ is positive definite if

(58) a>0  ⟹λmin{Kp}>kh2,α<b2c⟹λmin{Kd+Fv}>kh1,α<ab2ab+d2,

while *Q*_*b*_ is positive definite if

(59) a>0  ⟹  λmin{Kp}>kh2,b>0  ⟹  λmin{Kd+Fv}>kh1,α>e2ab.

Therefore, the selection of large enough control gains Δ, *K*_*p*_ and *K*_*d*_ guarantee the existence of *α* satisfying (43) so that the function $U\left(t,e,\dot{e}\right)$ in (45) is positive definite and radially unbounded, and $\dot{U}\left(t,e,\dot{e}\right)$ in (48) is a negative semidefinite function. Hence the trajectories **e**(*t*), $\dot{e}(t)$, $\tilde{W}(t)$, and $\tilde{V}(t)$ of the overall closed-loop system (40)-(32) are uniformly bounded.

By integrating both sides of (51) it is possible to prove that

(60) $$\begin{array}{c} \overset{t}{\underset{0}{\int }}{||p_{1}(\rho )||}^{2}d\rho \leq \frac{U\left(0,e\left(0\right),\dot{e}\left(0\right)\right)}{\lambda_{\min}\left\{Q_{1}\right\}}, \end{array}$$

where **p**_1_(*t*) is defined in (52). Then, by invoking Barbalat's lemma [31], the limit (44) is assured.

## 4. Experimental Results

Two sets of experiments in different experimental set-ups have been carried out in order to assess the performance of the proposed adaptive neural network controller (28) and (32)-(33). The used experimental systems are

- a horizontal two-degree-of-freedom arm,
- a vertical one-degree-of-freedom arm, which is affected by the gravitational force.

In each set of experiments the applied torque (28) is implemented with and without adaptive neural network compensation. The purpose of the experiments is to show the benefit of the adaptive neural network feedforward compensation.

### 4.1. Planar Two-Degree-of-Freedom Robot

**Figure 1:**
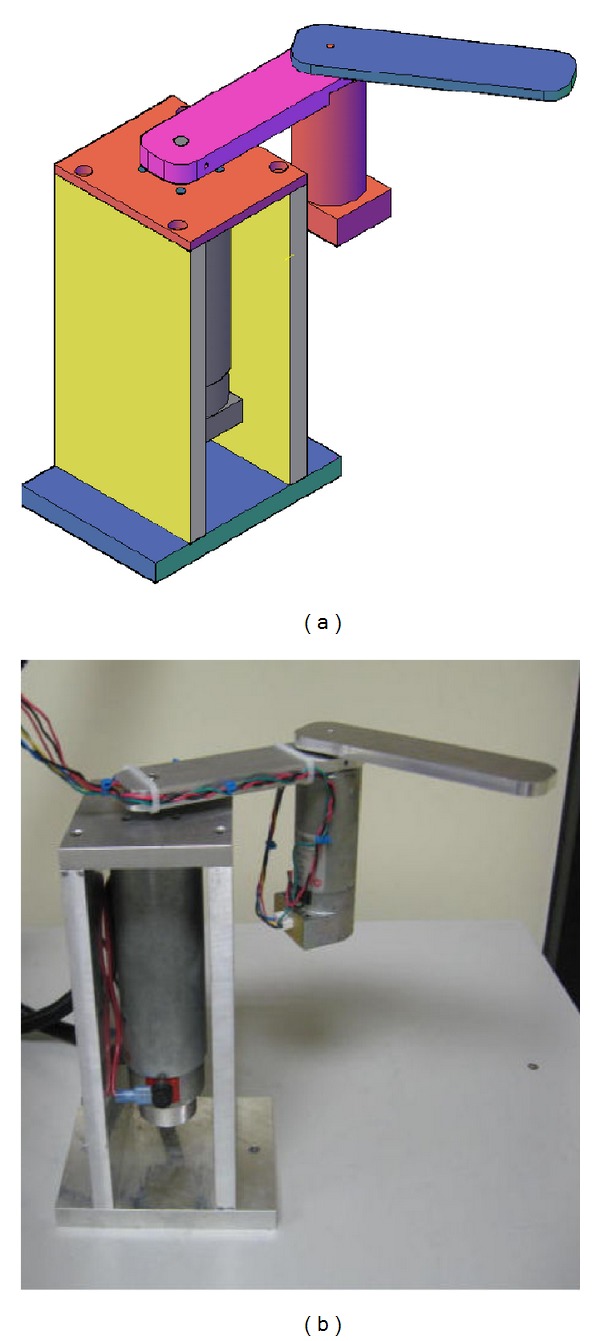
Experimental horizontal two-degree-of-freedom robot manipulator.

To carry out experiments, a planar two-degree-of-freedom direct-driven arm has been used. See Figure 1 for a CAD drawing and picture. The system is composed by two DC *Pittman* motors operated in current mode with two *Advanced Motion Controls* servo amplifiers. A *Sensoray 626* I/O card is used to read encoder signals with quadrature included and to transfer control commands through the D/A channels. A PC running *Windows XP*, *Matlab*, *Simulink,* and *Real-Time Windows Target* is used to execute controllers in real time at 1 [kHz] sampling rate.

Two experiments have been conducted corresponding to the implementation of a nonlinear PD controller which does not consider any adaptation and the proposed adaptive neural network-based controller in (28).

In reference to Tables 1 and 2, and the model (13), the elements of the experimental robot model are

(61) $$\begin{array}{c} M\left(q\right)=\left[\begin{array}{cc} \theta_{1}+2\theta_{2}\cos\left(q_{2}\right) & \theta_{3}+\theta_{2}\cos\left(q_{2}\right)\\ \theta_{3}+\theta_{2}\cos\left(q_{2}\right) & \theta_{3} \end{array}\right],\\ C\left(q,\dot{q}\right)=\left[\begin{array}{cc} -\theta_{2}\sin\left(q_{2}\right){\dot{q}}_{2} & -\theta_{2}\sin\left(q_{2}\right)\left[{\dot{q}}_{1}+{\dot{q}}_{2}\right]\\ \theta_{2}\sin\left(q_{2}\right){\dot{q}}_{1} & 0 \end{array}\right],\\ F_{v}=\mathrm{diag}\left\{\theta_{4},\theta_{5}\right\},\\ g\left(q\right)=0. \end{array}$$

It is noteworthy that the robot is moving in the horizontal plane, whereby the vector of gravitational forces is null.  Table 2 shows a numerical estimation of the parameters *θ*_*i*_ ∈ ℝ, which was obtained by using the filtered dynamic model and the classical least-squares identification method; see [32, 33]. The links of the arm are made of aluminum and both have a length of 0.15 [m].

#### 4.1.1. Desired Trajectory

The desired joint velocity trajectory chosen to achieve the experimental tests is given by

(62) $$\begin{array}{c} q_{d}\left(t\right)=\left[\begin{array}{c} 1.0\cos\left(2.0t\right)\\ 1.0\cos\left(4.0t\right) \end{array}\right]. \end{array}$$

**Figure 2:**
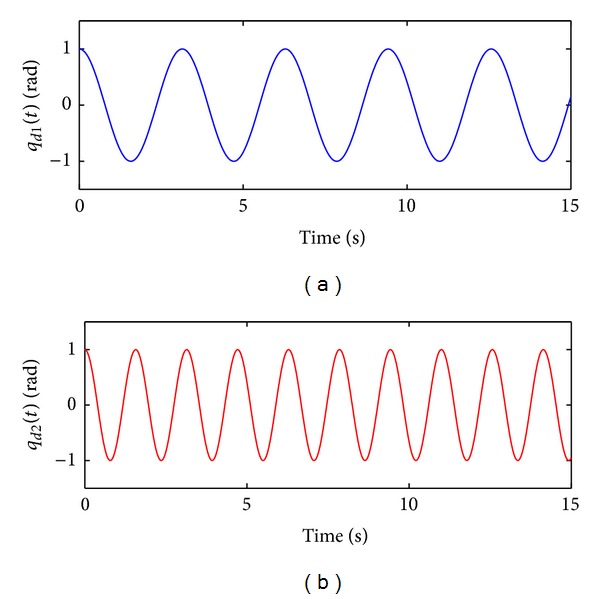
Desired joint trajectories *q*_*d*1_(*t*) and *q*_*d*2_(*t*) used in the real-time evaluations.

It is clear that the desired joint trajectory **q**_*d*_(*t*) satisfies the bounding assumption (22). See Figure 2 to observe the time evolution of the desired trajectory (62).

#### 4.1.2. Results

By removing the adaptive neural network compensation in the proposed controller (28), the following nonlinear PD-type controller is obtained:

(63) $$\begin{array}{c} \tau =K_{p}\tanh\left(\gamma e\right)+K_{d}\dot{e}+\Delta \mathrm{sign}\left(r\right), \end{array}$$

with **r** defined in (29). Hereafter, the controller (63) will be denoted as PD + Δsign(**r**).

The PD + Δsign(**r**) controller in (63) has been implemented in real time with the following control gains:

(64) Kp=diag{0.2,0.1},Kd=diag{0.1,0.01},Δ=diag{0.05,0.005},

and with

(65) $$\begin{array}{c} \alpha =1.0,\;\;\gamma =5.0. \end{array}$$

**Figure 3:**
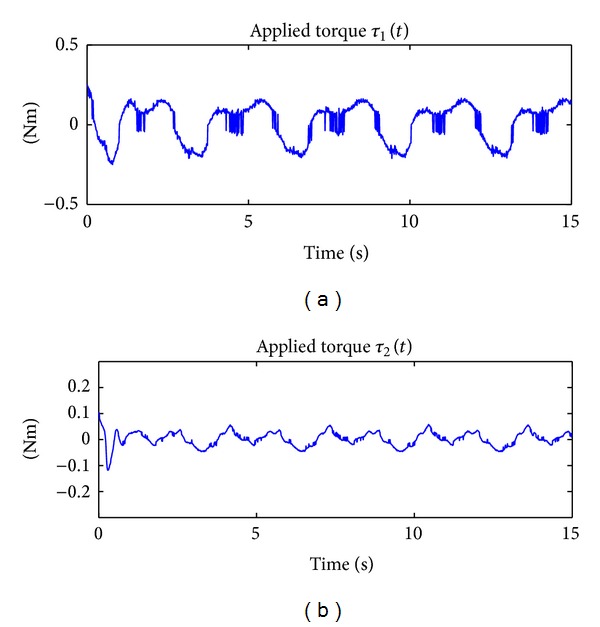
Applied torque by using the PD + Δsign(**r**) scheme which does not consider any adaptation.

**Figure 4:**
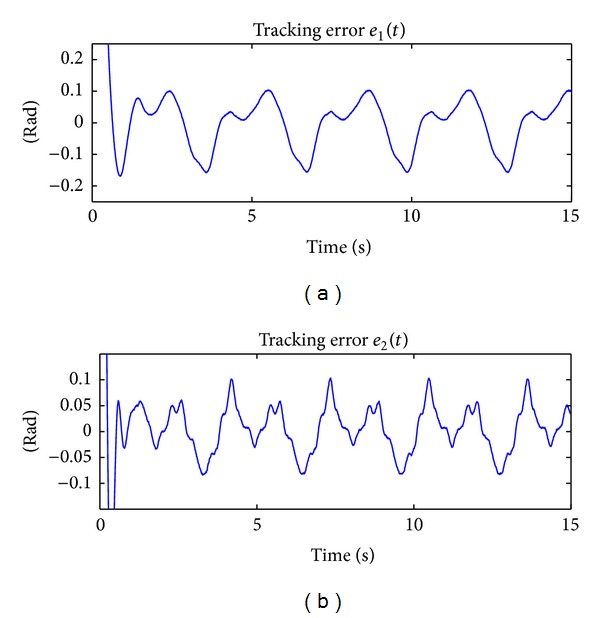
Tracking errors *e*_1_(*t*) and *e*_2_(*t*) by using the PD + Δsign(**r**) scheme which does not consider any adaptation.

The results of implementing in real time the PD + Δsign(**r**) controller in (63) can be appreciated in Figure 3, which shows the time evolution of the applied torques *τ*_1_(*t*) and *τ*_2_(*t*), and in Figure 4, that depicts the time evolution of the tracking error *e*_1_(*t*) and *e*_2_(*t*).

On the other hand, the new control scheme in (28) will be referred to as ANN + PD + Δsign(**r**). This controller was implemented by using *L* = 20, which is the number of neurons. The neural network requires the input vector

(66) $$\begin{array}{c} x_{d}={\left[q_{d1}\;{\dot{q}}_{d1}\;{\ddot{q}}_{d1}\;q_{d2}\;{\dot{q}}_{d2}\;{\ddot{q}}_{d2}\;1\right]}^{T}\in {\mathbb{R}}^{7}. \end{array}$$

The vector **x**_*d*_(*t*) describes the signals that are used in the inner layer of the neural network.

In order to establish a fair comparison scenery, the control gains (64) and (65), which were used in the PD + Δsign(**r**) controller in (63), have been used in the real-time implementation of the proposed controller ANN + PD + Δsign(**r**) in (28). Besides, the gains

(67) R=0.2I7,  F=0.5I20,

with *I*_*n*_ meaning the identity matrix of dimension *n*, were used the adaptation update laws (32) and (33), respectively.

**Figure 5:**
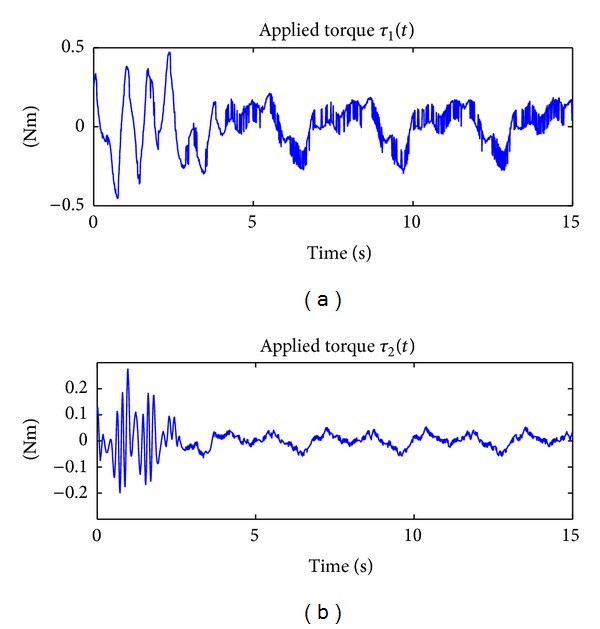
Applied torque by using the ANN + PD + Δsign(**r**) scheme which considers adaptation.

**Figure 6:**
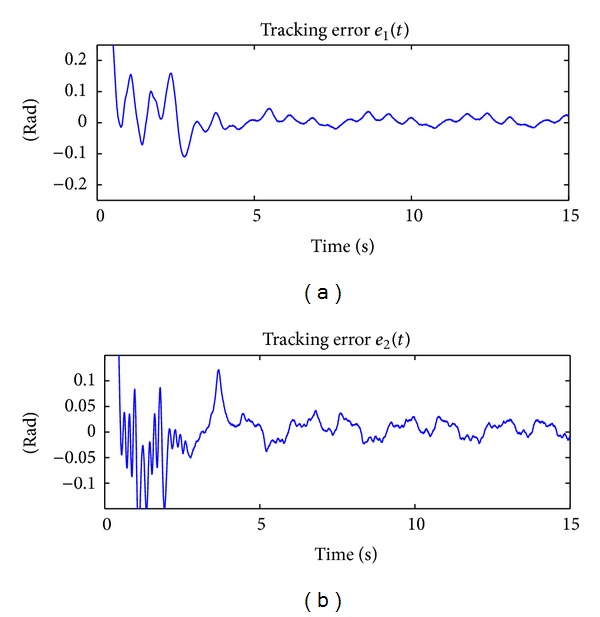
Tracking errors *e*_1_(*t*) and *e*_2_(*t*) by using the ANN + PD + Δsign(**r**) scheme which considers adaptation.

The results of implementing the proposed controller ANN + PD + Δsign(**r**) in (28) is shown in Figure 5, where the time evolution of the applied torques *τ*_1_(*t*) and *τ*_2_(*t*) are represented, and in Figure 6, where the obtained tracking errors *e*_1_(*t*) and *e*_2_(*t*) are described.

#### 4.1.3. Observations

Figures 4 and 6 show the tracking error **e**(*t*) ∈ ℝ^2^ for the controller PD + Δsign(**r**) in (63), which does not consider adaptation, and the new scheme ANN + PD + Δsign(**r**) in (28), which is based on the adaptation of neural network. From these figures the performance of the controllers can be assessed.

For both implementations, one can consider a steady state behavior after 10 [sec]. In order to assess the performance of the controllers, the maximum peak-to-peak tracking error for each joint and for each implementation has been computed.  Table 3 shows the performance of the two controllers, where the notation PTPV stands for “peak-to-peak value.” We can see that by using the new controller the tracking performance is drastically improved. In particular, the percentage of improvement for joint 1 is 82.3% and for joint 2 is 74.9%.

**Figure 7:**
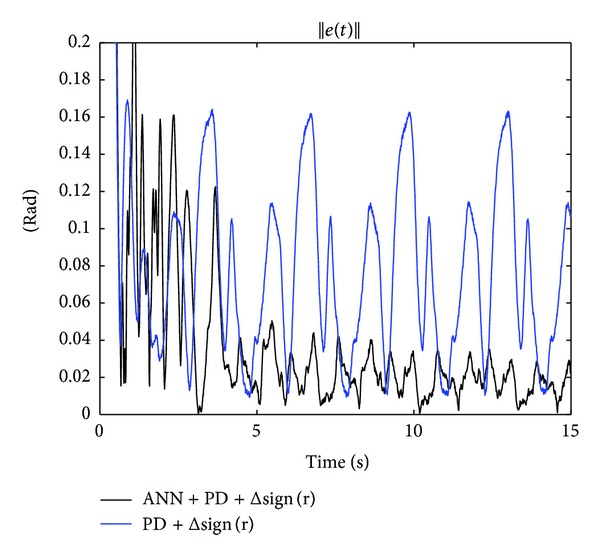
Time evolution of the norm of the tracking error ||**e**(*t*)||.

On other hand, Figure 7 shows the norm of the tracking error ||**e**(*t*)||, which is another form to compare the tracking performance of a controller with respect to the other. In this figure, the improvement in the tracking of the desired trajectory by using the ANN + PD + Δsign(**r**) scheme in (28) is clearly observed. After 10 [sec], the peak value of the ||**e**(*t*)|| by using the PD + Δsign(**r**) scheme in (63) is 0.161 [rad] and by using the ANN + PD + Δsign(**r**) controller in (28) is 0.035  [rad]. Thus, the improvement using the new scheme is 78.3%.

**Figure 8:**
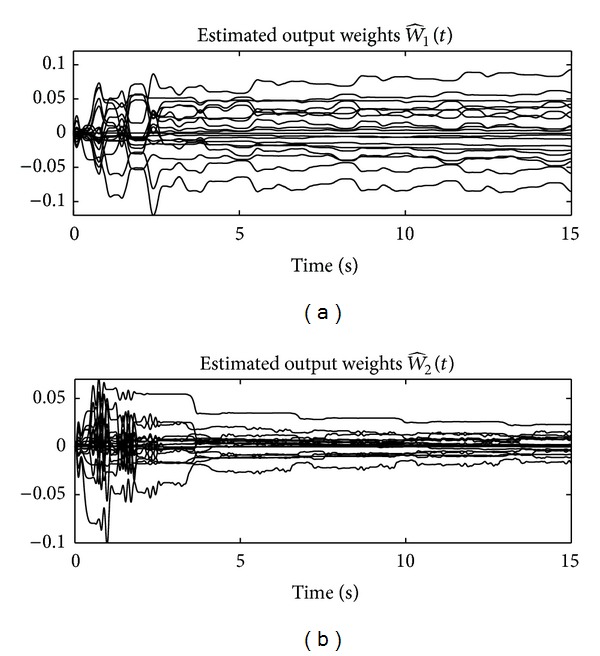
Time evolution of the output weights ${\hat{W}}_{1}\left(t\right)\in {\mathbb{R}}^{10\times 1}$ and ${\hat{W}}_{2}\left(t\right)\in {\mathbb{R}}^{10\times 1}$.

Finally, the output weights ${\hat{W}}_{1}(t)\in {\mathbb{R}}^{10\times 1}$ and ${\hat{W}}_{2}(t)\in {\mathbb{R}}^{10\times 1}$ for joints 1 and 2, respectively, obtained in the real-time implementation of the new controller (28) are observed in Figure 8. A small value of such a weights is enough to improve the tracking performance drastically.

### 4.2. Vertical One Degree-of-Freedom Robot

**Figure 9:**
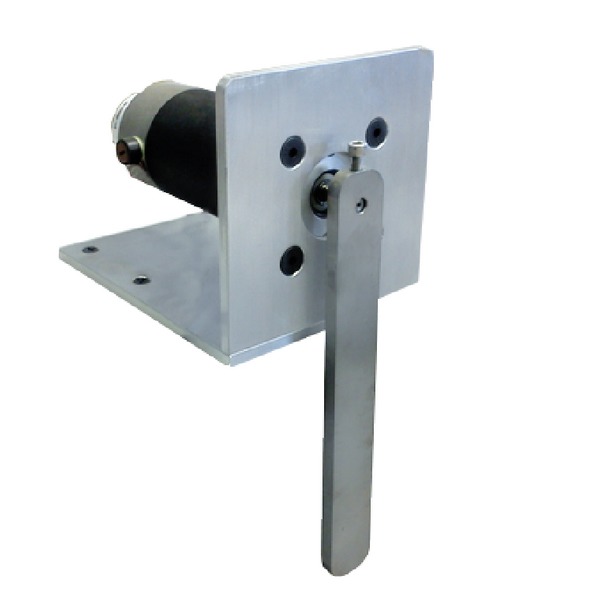
Experimental one-degree-of-freedom arm (pendulum) subject to gravitational force.

This experimental system consists in an *Advanced Motion Controls* servo amplifier operated in voltage mode and a *Nema 34* brushed direct current motor which has attached a pendulum as shown in Figure 9. Like the two-degree-of-freedom robot, a data acquisition board and Matlab were used to implement the controllers in real time at 1 [kHz] sampling rate.

The model of this system corresponds to a pendulum with viscous friction which is actuated by a armature-controlled direct current motor, which can be written as [34]

(68) $$\begin{array}{c} M\ddot{q}+B\dot{q}+N\sin\left(q\right)=I,\\ L\frac{dI}{d\text{t}}+RI+k_{B}\dot{q}=v, \end{array}$$

where *M*, *B*, *N*, *L*, *R*, and *k*_*B*_ are strictly positive constants; *I* denotes the armature current, and *v* is the voltage input. By using the least-squares identification method we have estimated the numerical value of the constant parameters, which are shown in Table 4. Let us notice that the motor shaft has attached a pendulum whose mass is 0.285 [Kg] and length is 0.1803 [m] (equivalent to 7.1 [in]) from the rotation axis to the arm tip.

The tested controllers corresponds to

(69) v=kptanh(γe)+kdq˙+δsign(r),

(70) v=W^Tσ(V^Txd)+kptanh(γe)+kdq˙+δsign(r).

In analogy with experimental tests carried out in the horizontal two-degree-of-freedom robot, the controller in (69) will be denoted as PD + *δ*sign(*r*), while the scheme (70) will be referred as ANN + PD + *δ*sign(*r*). In both controllers $r=\dot{e}+\alpha \tanh(\gamma e)$. The adaptation signals $\hat{V}(t)$ and $\hat{W}(t)$ in (70) are computed by using the expressions (32) and (32), respectively.

The idea is to compare the performance and robustness of the controllers (69) and (70) in a system affected by the gravitational force and the electrical dynamics of the motor.

In this case, the model of the system of the robot was assumed to be unknown, whereby the tested controllers were tuned by trial and error until an acceptable performance was obtained.

#### 4.2.1. Desired Trajectory

The proposed desired trajectory for this set of experiments was

(71) $$\begin{array}{c} {\text{q}}_{d}\left(t\right)=1.5+2\sin\left(3t\right)\;\left[\text{rad}\right]. \end{array}$$

**Figure 10:**
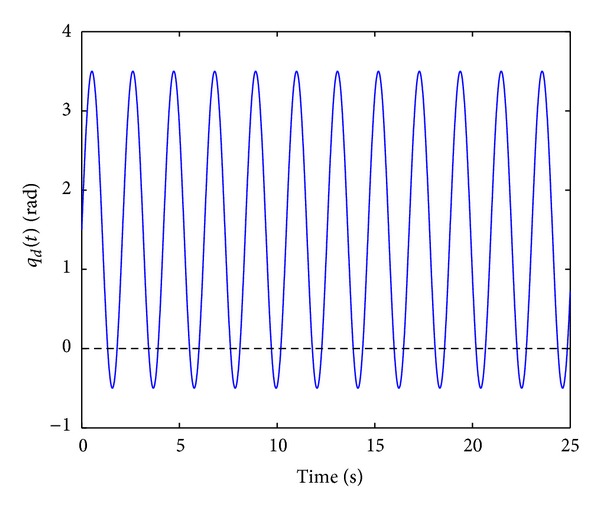
Desired joint trajectory *q*_*d*_(*t*) the real-time evaluations.

Notice that with trajectory the effect of the gravitational force is maximized.  Figure 10 shows the time history of the desired joint trajectory *q*_*d*_(*t*) in (71).

#### 4.2.2. Results

Like the case of the experiments with the two-degree-of-freedom robot, we have selected the control gains so that most of the control action is contributed by the adaptive neural network part of the control.

Specifically, we selected the gains

(72) kp=1,  kd=0.2,α=1.5,  γ=5.

The adaptation of the parameters is given by using (32) and (33) with $x_{d}={\left[1\;q_{d}\;{\dot{q}}_{d}\;{\ddot{q}}_{d}\right]}^{T}\in {\mathbb{R}}^{4}$ and the gains

(73) $$\begin{array}{c} R=0.2I_{4},\;\;F=0.3I_{10}. \end{array}$$

The proposed adaptive NN controller ANN + PD + *δ*sign(*r*) was implemented by using *L* = 10 neurons.

**Figure 11:**
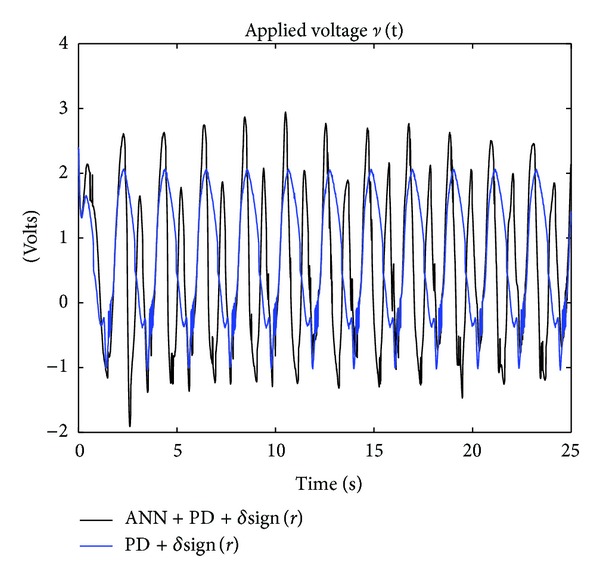
Experiments with the one degree-of-freedom robot: applied voltage *v*(*t*) for the PD + *δ*sign(*r*) and the ANN + PD + *δ*sign(*r*) controllers.

**Figure 12:**
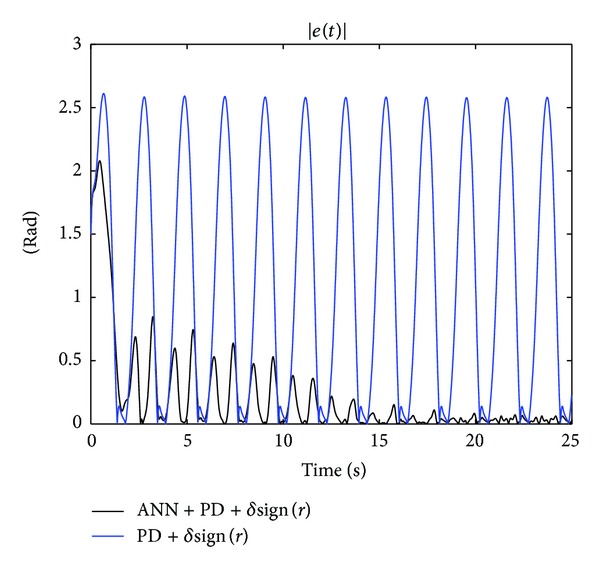
Experiments with the one degree-of-freedom robot: absolute value of tracking error |*e*(*t*)| for the PD + *δ*sign(*r*) and the ANN + PD + *δ*sign(*r*) controllers.

**Figure 13:**
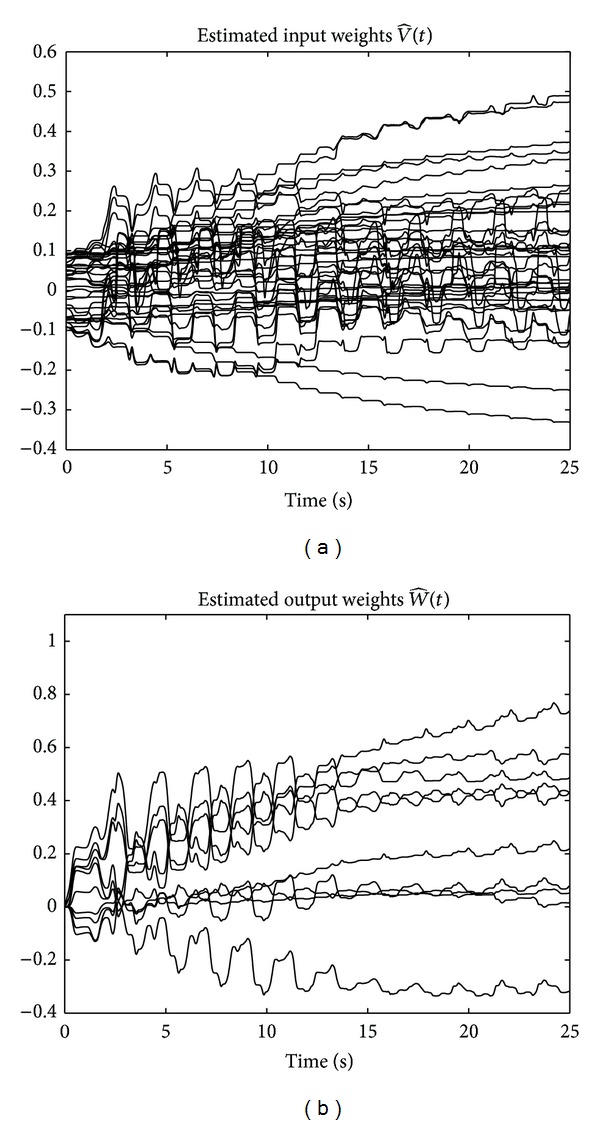
Experiments with the one degree-of-freedom robot: estimated input and output weights for the ANN + PD + *δ*sign(*r*) controller.

The results of the implementation of the controllers PD + *δ*sign(*r*) in (69) and ANN + PD + *δ*sign(*r*) in (70) are given in Figures 11 and 12, which show the time evolution of the applied voltage *v*(*t*) and the absolute value of tracking error |*e*(*t*)|, respectively. The advantage of the proposed controller in ANN + PD + *δ*sign(*r*) is clearly observed from Figure 12. Notice that with the new controller (70), which includes the adaptive neural network, the absolute value of the tracking error |*e*(*t*)| is reduced to values less than 0.08 [rad] after 20 [sec]. Finally, Figure 13 depicts the estimated input and output weights, $\hat{V}(t)$ and $\hat{W}(t)$, respectively, which remain bounded for all time.

## 5. Conclusions

This paper introduced a new adaptive neural network control algorithm for the tracking control of robot manipulators. The neural network of this controller uses only the desired joint position, velocity, and acceleration. The main theoretical result consisted in showing that the position and velocity error converge asymptotically while the input and output weights of the neural network remain bounded.

The experimental tests showed the benefit of using an adaptive neural network plus nonlinear PD control action. The new controller showed robustness to model uncertainties and strong nonlinear effects such as gravitational effects and Coulomb friction.

Further research considers the application of the proposed ideas in PI joint velocity control [35], anticontrol of chaos [36], output feedback tracking control [37], and underactuated systems [38].

## Figures and Tables

**Table 1:** Physical parameters of the experimental robot arm.

Description	Notation	Units
Length of link 1	*l*_1_	m
Length of link 2	*l*_2_	m
Distance to the center of mass of link 1	*l*_*c*1_	m
Distance to the center of mass of link 2	*l*_*c*2_	m
Mass of link 1	*m*_1_	kg
Mass of link 2	*m*_2_	kg
Inertia rel. to center of mass (link 1)	*I*_1_	kg-m^2^
Inertia rel. to center of mass (link 2)	*I*_2_	kg-m^2^

**Table 2:** Estimated parameters of the experimental robot arm; see (61) for reference.

Parameter	Definition	Value	Unit
*θ*_1_	*m*_1_ *l*_*c*1_^2^ + *m*_2_[*l*_1_^2^ + *l*_*c*2_^2^] + *I*_1_ + *I*_2_	0.0363	Kg m^2^
*θ*_2_	*m*_2_ *l*_1_ *l*_*c*2_	0.0028	Kg m^2^
*θ*_3_	*m*_2_ *l*_*c*2_^2^ + *I*_2_	0.0023	Kg m^2^
*θ*_4_	*f*_*v*1_	0.0084	Nm sec
*θ*_5_	*f*_*v*1_	0.0024	Nm sec

**Table 3:** Performance of the two controllers: maximum peak-to-peak value (PTPV) of the tracking errors after the settling time.

Index [rad]	PD + Δsign(r)	ANN + PD + Δsign(r)
max_*t*≥10_{PTPV of *e*_1_(*t*)} (rad)	0.260	0.046
max_*t*≥10_{PTPV of *e*_2_(*t*)} (rad)	0.187	0.047

**Table 4:** Identified parameters of the vertical one degree-of-freedom robot.

Parameter	Unit	Estimation
Lumped inertia *M*	A(s^2^/rad)	0.0292
Lumped viscous friction coefficient *B*	A(s/rad)	0.0298
Lumped gravitational load *N*	A	2.2387
Inductance *L*	H	0.0031
Resistance *R*	Ω	0.9322
Back electromotive force coefficient *k*_*B*_	N(m/A)	0.0246
